# Efficacy and safety of neuromodulation and multimodal therapies for traumatic brain injury-induced disorders of consciousness: an updated umbrella review

**DOI:** 10.3389/fneur.2026.1742096

**Published:** 2026-03-04

**Authors:** Xia Yang, Yongbiao Li, Nana Zhang, Dongwei Luo, Chunying Zhao, Qingshan Liu

**Affiliations:** 1 Key Laboratory of Ethnomedicine of Ministry of Education, School of Pharmacy, Center on Translational Neuroscience, Minzu University of China, Beijing, China; 2 Department of Respiratory and Critical Care Diseases, Senior Department of Infectious Diseases, The Fifth Medical Center of Chinese PLA General Hospital, Beijing, China

**Keywords:** disorders of consciousness, neuromodulation, neurorehabilitation, transcranial magnetic stimulation, traumatic brain injury

## Abstract

**Background:**

Post-traumatic disorders of consciousness (DoC) remain a major barrier to recovery after traumatic brain injury (TBI), yet therapeutic guidance is fragmented across modalities.

**Objective:**

To synthesize the highest-level evidence on efficacy and safety of interventions for TBI-related DoC and derive practice-oriented recommendations.

**Methods:**

Following PRISMA and a prospectively registered protocol (INPLASY202480015), we systematically screened PubMed, Embase, Web of Science, and CNKI through June 2024 for peer-reviewed systematic reviews and meta-analyses focused on TBI-induced DoC. Methodological quality was appraised using AMSTAR-2. Primary outcomes were CRS-R, GCS, GOS, and overall efficacy rate; random- or fixed-effects models were applied per heterogeneity.

**Results:**

Seven high-quality evidence syntheses encompassing 121 trials and eight interventions were included. Neuromodulation showed consistent benefits: repetitive transcranial magnetic stimulation (rTMS) improved CRS-R (MD 3.00, 95% CI 2.47–3.52) and GCS (MD 2.92, 1.65–4.19); transcranial direct current stimulation (tDCS) improved CRS-R (MD 2.08, 0.63–3.25). Peripheral and sensory approaches were robust: acupuncture improved GCS (MD 2.03, 1.54–2.52), GOS (RR 1.22, 1.16–1.29), and Efficacy Rate (RR 1.48, 1.40–1.56); multisensory stimulation improved GCS (MD 2.28, 2.02–2.54) and GOS (MD 1.11, 0.77–1.45). Right median nerve stimulation (RMNS) and family-centered sensory-affective stimulation also yielded significant gains, while single-study Trigeminal nerve stimulation (TNS) effects were mixed.

**Conclusions:**

tDCS, rTMS, median nerve stimulation, multisensory stimulation, and acupuncture emerge as leading strategies for TBI-related DoC. We highlight priorities for the field: adequately powered multicenter RCTs with standardized protocols, mechanistic studies to refine dosing and targets, and predictive tools for personalized therapy selection. This umbrella synthesis provides a pragmatic evidence map to accelerate recovery and improve long-term outcomes in this vulnerable population.

## 1 Introduction

Traumatic Brain Injury (TBI) is a neurological disorder resulting from external mechanical forces that induce structural and functional brain damage (1). As one of the most common neurological conditions worldwide, TBI represents a growing public health challenge with substantial socioeconomic implications (2, 3). The pathophysiology of TBI involves a dynamic interplay between primary mechanical injury and subsequent secondary injury cascades, culminating in transient or persistent neurological impairments (4). Global epidemiological data demonstrate that there were 20.84 million incident cases and 37.93 million prevalent cases of TBI worldwide in 2021. Notably, age-standardized incidence, prevalence, and disability-adjusted life years (DALYs) related to TBI have decreased by approximately 20% since 1990, which reflects substantial advances in TBI prevention and clinical care. (1, 5, 6). While advancements in neurosurgical techniques and neurocritical care have markedly reduced TBI-associated mortality, a considerable proportion of survivors experience debilitating long-term sequelae, including Disorders of Consciousness (DoC) (7, 8).

DoC encompasses a spectrum of conditions characterized by impaired arousal and awareness, classified into three primary states: coma, unresponsive wakefulness syndrome (UWS), andminimally conscious state (MCS) (8, 9). Coma is defined by the absence of both spontaneous arousal and stimulus-induced wakefulness. UWS (previously termed vegetative state) describes a condition in which patients exhibit wakefulness without evidence of conscious awareness. In contrast, MCS is distinguished by inconsistent but discernible behavioral signs of awareness, indicating either partial recovery from severe brain injury or progressive neurological deterioration (10). These disorders profoundly diminish patients' quality of life while imposing immense psychological and economic strain on caregivers and healthcare systems. Consequently, the development of effective therapeutic strategies remains a critical priority in neurorehabilitation, with significant clinical and societal implications.

Neuromodulation therapies, defined as non-invasive or minimally invasive interventions that regulate neural electrical activity, synaptic plasticity, or nerve conduction to promote consciousness recovery, have emerged as an innovative and promising therapeutic strategy in restorative medicine for TBI. (11)Among invasive techniques, deep brain stimulation (DBS) has been experimentally shown to reconstruct neural connectivity and sustain neuronal activity in the forebrain, with established clinical applications in movement disorders such as Parkinson's disease and essential tremor (12). However, its widespread adoption in TBI management remains limited due to inherent surgical risks, demanding postoperative care, and high treatment costs (13, 14). In recent years, the field of clinical intervention for DoC after TBI has attracted considerable attention, with safe and practical non-invasive brain stimulation (NIBS)-based neuromodulation therapies and multimodal rehabilitation interventions emerging as mainstream and widely recognized approaches (8, 15). Among them, neuromodulation therapies are classified into central neuromodulation [targeting the cerebral cortex, including repetitive transcranial magnetic stimulation (rTMS) and transcranial direct current stimulation (tDCS)] and peripheral neuromodulation [targeting peripheral nerves, including median nerve stimulation (MNS), right median nerve stimulation (RMNS), and trigeminal nerve stimulation (TNS)] based on the stimulation site, primarily functioning by regulating cortical excitability and neural activity (16, 17). In addition to neuromodulation therapies, multimodal rehabilitation interventions—defined as non-pharmacological, comprehensive interventions that improve consciousness and cognitive function through multiple action pathways (excluding neuromodulation)—have also been proven effective in improving the consciousness state (18–20).

TMS non-invasively induces neuronal electrical activity through rapidly alternating magnetic fields, whereas tDCS modulates neuronal firing thresholds via low-intensity direct current application (21, 22). Clinical evidence suggests that both tDCS and TMS can effectively enhance consciousness levels in post-TBI comatose patients, leading to their increasing integration into clinical practice (23). Additionally, MNS exerts its effects by modulating electrophysiological signals transmitted from peripheral nerves to the central nervous system, demonstrating considerable therapeutic potential (24). Preliminary findings indicate that RMNS may exhibit superior arousal-promoting effects, though further clinical validation is warranted (25, 26). Similarly, TNS facilitates consciousness recovery by activating neuronal circuits in the lateral hypothalamus and spinal trigeminal nucleus, positioning it as a highly promising intervention for coma arousal (27).

Multimodal rehabilitation interventions can be divided into two major categories: first, sensory stimulation interventions, which promote cortical activation via multi-dimensional sensory input and include Multi-sensory stimulation and family-centered sensory and affective stimulation. Multi-sensory stimulation accelerates the awakening of comatose patients by integrating visual, tactile, olfactory, and other sensory inputs (19, 20). Family-mediated sensory and affective stimulation not only enhances consciousness but also promotes the recovery of cognitive and basic sensory functions, highlighting its clinical significance (28). The second category is traditional Chinese medicine (TCM)-derived therapy based on TCM theory, such as Acupuncture (18). In this study, multimodal rehabilitation interventions were included on the premise that they target DoC induced by TBI, have complete efficacy data from clinical studies, and achieve consciousness recovery through non-neuromodulatory mechanisms.

Numerous systematic reviews and meta-analyses have investigated therapeutic interventions for DoC secondary to TBI. Nevertheless, current evidence remains limited by several methodological shortcomings, including substantial heterogeneity in study designs, inadequate sample sizes, and inconsistent outcome measurement criteria. To address these limitations, this study proposes to perform a comprehensive evidence synthesis through a systematic evaluation of the most recent literature comprising systematic reviews and meta-analyses on TBI-induced DoC treatments. The study has three primary objectives: 1) to systematically assess the efficacy and safety profiles of existing therapeutic modalities; 2) to conduct comparative analyses of clinical outcomes across different treatment regimens; and 3) to formulate evidence-based clinical practice recommendations. By implementing this rigorous methodological approach, we intend to identify optimal treatment strategies for post-TBI DoC patients, with the ultimate goal of enhancing clinical outcomes and advancing patient care standards.

## 2 Materials and Methods

This study was conducted in accordance with the Preferred Reporting Items for Systematic Reviews and Meta-Analyses (PRISMA) guidelines (29). The study protocol was prospectively registered on the INPLASY PROTOCOL platform (Registration No:INPLASY202480015) (30). Briefly, we systematically searched four major databases from inception to December 2025, supplemented with manual searches and reference tracing, to identify eligible systematic reviews, meta-analyses, and clinical trials focusing on clinical assessment scales for TBI-induced DoC. Studies were screened based on predefined inclusion and exclusion criteria, and methodological quality was evaluated using the AMSTAR 2 tool. Statistical analyses were performed using mean differences (MD) or risk ratios (RR) with 95% confidence intervals (95% CI); random-effects or fixed-effects models were selected based on heterogeneity assessed by the *I*^2^ statistic, aiming to evaluate the efficacy of the intervention on DoC recovery via four primary outcome measures.

### 2.1 Search strategy and quality assessment

We conducted a comprehensive systematic search across four major databases–CNKI, PubMed, Embase, and Web of Science–to identify all relevant Chinese and English literature published from database inception through December 2025. Gray literature and unpublished studies were excluded. Keywords including Brain injury, Consciousness Disorders, systematic review, meta-analysis, clinical trial were adopted. Boolean operators (AND/OR) were systematically applied to construct logical search strings. This approach was designed to guarantee the comprehensiveness of search results and consistency across different databases. In addition, manual searches and reference tracing were conducted to supplement the electronic search, further ensuring the comprehensiveness of the included literature.

Studies were included if they met the following criteria: 1) Published in English or Chinese in peer-reviewed journals; 2) Reported as systematic reviews or meta-analyses with publicly accessible data; 3) Focused on clinical assessment scales for disorders of consciousness (DoC); 4) Specifically addressed TBI-induced DoC. Exclusion criteria comprised:1)Unpublished or non-peer-reviewed studies; 2) Studies with insufficient methodological detail or incomplete outcome data; 3) Investigations of DoC secondary to non-traumatic etiologies (e.g., hypoxic encephalopathy, metabolic disorders).

The methodological quality of the included systematic reviews and meta-analyses was evaluated using the AMSTAR2 (A MeaSurement Tool to Assess systematic Reviews 2) instrument (31). This tool assesses research quality across 16 evaluation items and categorizes it into four tiers: High, Moderate, Low, and Critically low. The classification is primarily based on the presence of critical and non-critical domains in the study. Critical domains refer to major limitations that directly compromise the reliability of study conclusions, such as the absence of prospective registration for the literature search strategy and failure to evaluate publication bias among included studies. In contrast, non-critical domains denote minor deficiencies that do not affect the core conclusions but may reduce the methodological rigor of the research, for instance, lack of dual independent data extraction. Studies were classified into four quality tiers based on the presence of critical and non-critical flaws: High (no or one non-critical weakness), Moderate (more than one non-critical weakness), Low (one critical flaw), and Critically Low (more than one critical flaw).

### 2.2 Data extraction

Two investigators (YX and LYB) independently performed data extraction using a standardized data collection form. Any discrepancies in extracted data were resolved through consensus discussions involving two senior researchers (ZNN, LDW). All collected data were subsequently consolidated into a comprehensive extraction database.

The key characteristics of included studies were systematically tabulated, including publication year, study design, sample size, and intervention protocols. Treatment efficacy was evaluated using at least one of the following validated clinical assessment tools: (1) Glasgow Coma Scale (GCS); (2) Glasgow Outcome Scale (GOS); (3) Revised Coma Recovery Scale (CRS-R); or (4) Efficacy rate.

The selection of appropriate assessment scales was guided by several methodological considerations, including study design characteristics, sample size, and effect size measures (mean differences or risk ratios, both reported with random effects and 95% confidence intervals). Heterogeneity was quantitatively assessed using the *I*^2^ statistic and interpreted according to conventional thresholds: 0–25% (minimal), 26–50% (moderate), and 51–75% (substantial). In accordance with established meta-analytic principles (25, 32), we employed a random-effects model when *I*^2^ values exceeded 50% to account for between-study variability; otherwise, a fixed-effects model was utilized. This analytical approach ensured appropriate handling of heterogeneity while maintaining statistical rigor.

### 2.3 Statistical analysis

To evaluate the efficacy of the investigational drug in improving disorders of consciousness following traumatic brain injury, this study employed four primary outcome measures: 1) Acute consciousness evaluation using GCS—the established gold standard for stratifying consciousness impairment in severe brain injury patients (33), which provides objective quantification of acute neurological deficits. Mean Difference (MD) is used to compare group differences; a clinically significant MD is typically set as >1 point for GCS; 2) Neurobehavioral monitoring via CRS-R, employing standardized behavioral paradigms (34, 35) to sensitively track consciousness recovery trajectories and enable diagnostic differentiation. MD is also applied here, with a clinical significance threshold often considered >2 points for CRS-R; 3) Long-term prognostic assessment with GOS as the predefined 3-month primary endpoint (36), a validated measure strongly associated with functional recovery in severe TBI populations. Both MD and Relative Risk (RR) can be used; an RR >1 indicates a higher probability of favorable outcome; 4) Efficacy rate serves as a composite indicator reflecting the proportion of patients achieving clinically meaningful improvements. In meta-analyses, it is commonly expressed as a relative risk (RR) or odds ratio (OR), quantifying the likelihood of therapeutic benefit in the intervention group relative to the control group (37). An RR > 1 indicates a higher likelihood of achieving this composite clinical improvement.

Treatment effects were quantitatively assessed by calculating MD or RR, each reported with corresponding 95% confidence intervals (95% CI) and *P*-values for statistical significance.

## 3 Results

### 3.1 Literature search and screening process

**Figure 1:**
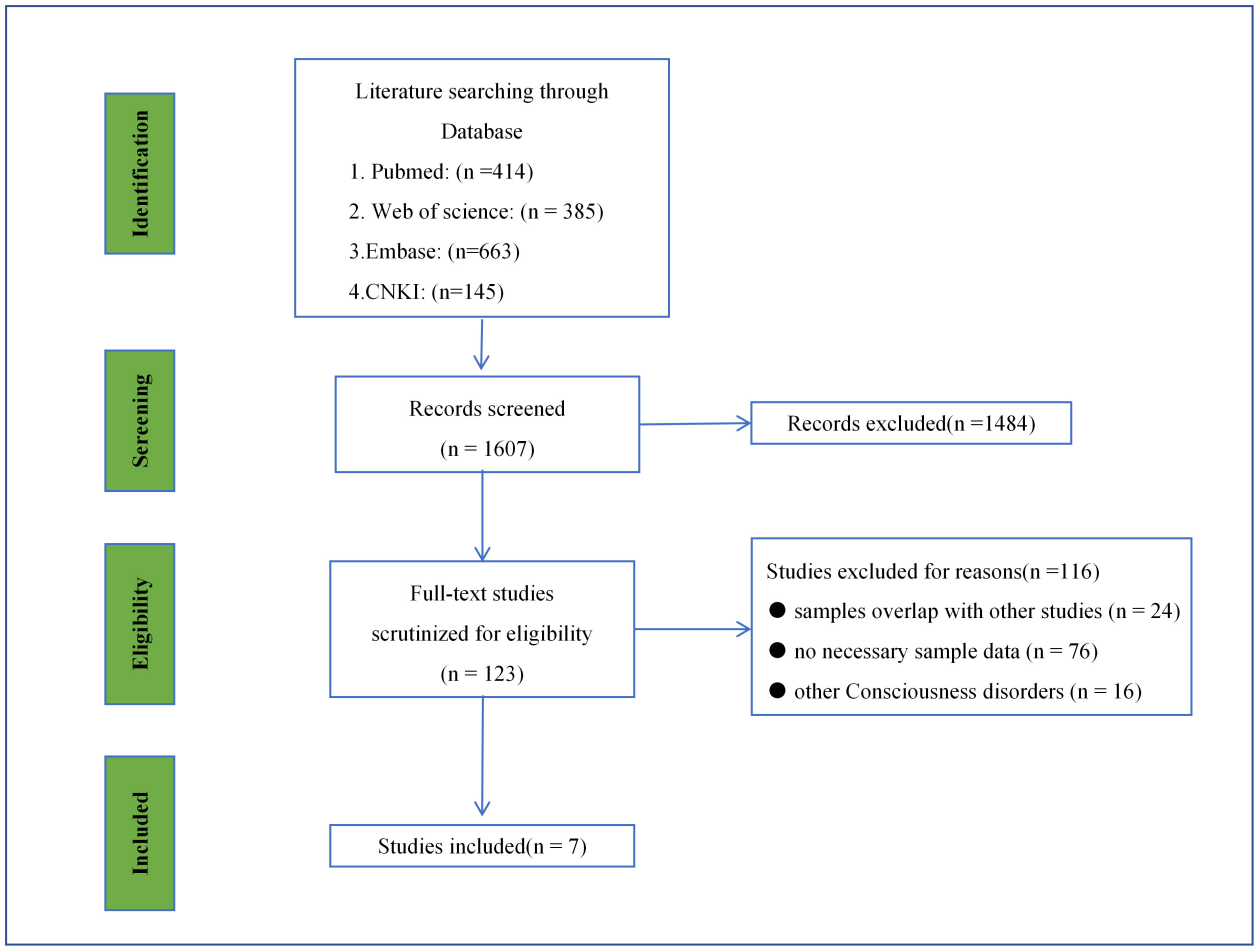
The search and screening process.

A comprehensive systematic search was conducted across four major academic databases (PubMed, Web of Science, Embase, and CNKI), identifying an initial pool of 1,607 potentially relevant publications. Following preliminary screening of titles and abstracts, 123 studies were selected for full-text assessment. During the rigorous full-text evaluation process, 116 studies were excluded based on predefined inclusion criteria. The exclusion criteria were applied as follows: duplicate sample data (*n* = 24), insufficient reporting of key outcome measures (*n* = 76), and inclusion of patients with consciousness disorders attributable to alternative etiologies (*n* = 16) (Figure 1). This rigorous selection process resulted in the inclusion of seven studies for final analysis (18, 20, 28, 32, 34, 38, 39).

As shown in Table 1, the included studies encompassed a total of 121 clinical trials, covering 8 distinct intervention modalities. These modalities are mainly classified into two categories: neuromodulation therapies (central neuromodulation: rTMS, tDCS; peripheral neuromodulation: RMNS, TNS, MNS) and multimodal rehabilitation interventions (sensory stimulation interventions, including family-centered sensory and affective stimulation, multi-sensory stimulation; traditional Chinese medicine (TCM)-derived therapy, including acupuncture). All the above three categories of interventions were included in the analysis after rigorous literature screening, which was consistent with the overall screening process described in the “Search Strategy” section. All studies meeting the inclusion criteria focused on DoC induced by TBI, with corresponding reports from systematic reviews, Meta analyses or RCTs and complete outcome data. This ensures that each intervention is highly relevant to the research topic and supported by reliable clinical evidence for subsequent efficacy analysis.

**Table 1:** Description and AMSTAR scores of included studies.

**No**	**Study**	**Conditions**	**Studies included**	**Study duration (median, range)**	**Daily dose (median, range)**	**Outcomes**	**AMSTAR2 scores**	**Study quality**
1	(18)	Acupuncture	49	unclear/ 4w(1W−12W)	/	GCS, GOS, Efficacy Rate	High	
2	(32)	rTMS	16	unclear/ 4W(2W−12W)	20min (0.83min−30min)	GCS, CRS–R	High	
	(32)	tDCS	12	10D(3D−28D)	2mA/4mA 20min	GCS, CRS–R		
3	(38)	RMNS	1	2W	8 h/day	GCS, CRS–R, Efficacy Rate	N/A	
4	(34)	TNS	1	4W	3 h/day	CRS–R, GCS	N/A	
5	(28)	family–centered sensory and affective stimulation	17	12D (1D−60D)	30min/time; 3times/day (10–60min/time; 1–8times/day)	GCS	Low	
6	(20)	Multi-sensory stimulation	13	unclear/ 30D(7D−90D)	/	GCS, GOS	Low	
7	(39)	MNS	12	4W(2W−12W)	/	GCS	Low	

All included studies enrolled patients aged ≥18years, and the types of consciousness disorders covered in these studies included coma, minimally conscious state (MCS), vegetative state (VS), locked-in state, and unresponsive wakefulness syndrome (UWS). All investigations employed randomized controlled trial designs, with intervention durations varying from 1 to 90 days. The methodological quality of the studies included in this review was systematically appraised with the AMSTAR−2 instrument. Findings revealed that the overall methodological quality was moderate, which was mainly constrained by the suboptimal literature search strategies and inadequate bias assessment in a proportion of the included studies. Accordingly, caution is warranted in the discussion and interpretation of these evaluation results.

### 3.2 GCS-R

Based on the meta-analysis of included studies (Table 2), three studies evaluated MD in GCS-R score changes from baseline across different interventions. The results demonstrated that both rTMS and tDCS were effective in improving disorders of consciousness.

**Table 2:** Results of pairwise meta-analyses for CRS-R.

**S. No**	**Comparative intervention**	**References intervention**	**Pairwise meta-analyses**						
			**Number of studies**	**Number of controls**	**Number of patients**	**MD/TD**	**95% CI**	***I* ^*2*^**	***p***
1	rTMS	Routine rehabilitation/ sham stimulation	14	300	306	MD= 3.00	(2.47, 3.52)	61%	*P* < 0.00001
2	tDCS	Routine rRehabilitation/ sham stimulation	12	224	224	MD = 2.08	(0.63, 3.25)	80%	*P* < 0.00001
3	TNS	The sham stimulation group	1	6	4	TD = 2.2	(-0.20, 4.5)	/	0.104

MD, mean difference; TD, treatment difference; CI, confidence interval; I^2^, heterogeneity.

Pooled data from two studies (66.7%) indicated that rTMS produced a significantly greater improvement in GCS-R scores compared to the control group (MD = 3.00, 95% CI: 2.47–3.52). Similarly, tDCS showed a clinically meaningful benefit (MD = 2.08, 95% CI: 0.63–3.25). In contrast, a single study (33.3%) assessing TNS reported a non-significant therapeutic effect (MD = 2.2, 95% CI:−0.2–4.5; *P*> 0.05). This analysis employed mean differences with 95% confidence intervals to evaluate the efficacy of neuromodulatory interventions in enhancing consciousness recovery.

### 3.3 GCS

Table 3 Results demonstrate consistent and statistically significant improvements in GCS scores across all six included studies (100%) when comparing neurostimulation interventions to conventional treatment or sham stimulation in patients with disorders of consciousness. The key findings are summarized as follows:

1. Acupuncture intervention: MD = 2.03 (95% CI: 1.54–2.52)
2. rTMS: MD = 2.92 (95% CI: 1.65–4.19)
3. TNS: MD = 2.40 (95% CI: 0.60–4.20)
4. Multisensory stimulation: MD = 2.28 (95% CI: 2.02–2.54)
5. Family-centered sensory-affective stimulatio: MD = 1.90 (95% CI: 1.69–2.12)
6. MNS: 4-week intervention: MD = 2.12 (95% CI: 0.99–3.24);8-week intervention: MD = 1.47 (95% CI: 0.46–2.48)

**Table 3:** Results of pairwise meta-analyses for GCS.

**S. No**	**Comparative intervention**	**References intervention**	**Pairwise meta-analyses**						
			**Number of studies**	**Number of controls**	**Number of patients**	**MD/TD**	**95% CI**	***I* ^*2*^**	***p***
1	Acupuncture	Basic treatment or combined with rehabilitation training	22	768	780	MD = 2.03	(1.54, 2.52)	78%	*P* < 0.00001
2	rTMS	Routine rehabilitation/sham stimulation	6	161	162	MD = 2.92	(1.65, 4.19)	95%	*P* < 0.00001
3	TNS	The sham stimulation group	1	6	4	TD = 2.4	(0.6, 4.2)	/	0.028
4	Family-centered sensory and affective stimulation	Routine care	11	274	280	MD = 1.90	(1.69, 2.12)	34%	*P* < 0.00001
5	Family-centered sensory and affective stimulation	Nurse's sensory stimulation	3	102	103	MD = 2.17	(1.67, 2.66)	0	*P* < 0.00001
6	Multi-sensory stimulation	Routine rehabilitation	10	355	360	MD = 2.28	(2.02, 2.54)	0	*P* < 0.00001
7	MNS	Basic treatment	8	392	397	MD = 2.12	(0.99, 3.24)	91%	0.0002
8	MNS	Basic treatment	4	106	106	MD = 1.47	(0.46, 2.48)	91%	0.004

MD, mean difference; TD, treatment difference; CI, confidence interval; I^2^, heterogeneity.

Notably, the family-centered sensory-affective stimulation therapy not only demonstrated significantly greater efficacy than conventional treatment (*P* < 0.05) but also outperformed the professional nurse care subgroup in terms of efficacy in the subgroup analysis. These findings underscore the potential of neurostimulation approaches in enhancing consciousness recovery, with varying degrees of efficacy across different modalities.

### 3.4 GOS

Table 4 compares the efficacy of two interventions —multi-sensory stimulation and acupuncture —in improving GOS scores among patients with disorders of consciousness. Pooled results from both included studies (100%) indicated that these interventions were significantly more effective than conventional therapy.

**Table 4:** Results of pairwise meta-analyses for GOS.

**S. No**	**Comparative intervention**	**References intervention**	**Pairwise meta-analyses**						
			**Number of studies**	**Number of controls**	**Number of patients**	**MD/RR**	**95% CI**	***I* ^2^**	***p***
1	Acupuncture	Basic treatment or combined with rehabilitation training	21	811	877	RR=1.22	(1.16, 1.29)	14%	*P* < 0.00001
2	Acupuncture	Basic treatment or combined with rehabilitation training	3	83	81	MD=0.77	(0.40, 1.13)	75%	*P* < 0.0001
3	Multi-sensory stimulation	Routine rehabilitation	3	84	84	MD= 1.11	(0.77, 1.45)	0%	*P* < 0.00001

MD, mean difference; RR, risk rate; CI, confidence interval; I^2^, heterogeneity.

Specifically, multi-sensory stimulation demonstrated a statistically significant therapeutic effect, (MD = 1.11, 95% CI: 0.77–1.45). Similarly, acupuncture treatment showed robust efficacy, supported by both MD and relative risk (RR) analyses (MD = 0.77, 95% CI: 0.40–1.13; RR = 1.22, 95% CI: 1.16–1.29).

### 3.5 Efficacy Rate

Table 5 presents the comparative results of different interventions in terms of Efficacy Rate. Both included studies (100%) demonstrated that acupuncture and RMNS were significantly superior to conventional treatment or combined rehabilitation training in improving patients' symptoms. Detailed analysis revealed that the Efficacy Rate in the acupuncture group was 1.48 times that of the control group (RR = 1.48, 95% CI: 1.40–1.56), while the hazard ratio (HR) for the RMNS group was 1.75 (95% CI: 1.30–2.34), both of which were statistically significant.

**Table 5:** Results of pairwise meta-analyses for Efficacy Rate.

**S. No**	**Comparative medications**	**References medications**	**Pairwise meta-analyses**						
			**Number of studies**	**Number of controls**	**Number of patients**	**RR/HR**	**95% CI**	***I* ^2^**	***p***
1	Acupuncture	Basic treatment or combined with rehabilitation training	34	1163	1214	RR = 1.48	(1.40, 1.56)	22%	*P* < 0.00001
2	RMNS	Routine treatment	1	162	167	HR = 1.75	(1.30, 2.34)	/	0.0002

RR, risk ratio; HR, Hazard ratio; CI, confidence interval; I^2^, heterogeneity.

## 4 Discussion

This systematic review evaluates the efficacy and safety of various treatment modalities for patients with DoC following TBI, synthesizing evidence from existing systematic reviews and meta-analyses to inform clinical decision-making.

Our comprehensive analysis demonstrates that several interventions —including tDCS, rTMS, MNS, multisensory stimulation, and acupuncture —significantly improve consciousness levels in TBI-induced DoC patients, corroborating findings from prior studies. The analysis confirms that tDCS, rTMS, MNS, and multisensory stimulation constitute robust therapeutic strategies, as evidenced by their consistent and statistically significant improvements across primary outcome measures including CRS-R, GCS, and GOS. Specifically, tDCS yielded notable improvements in CRS-R scores (MD 2.08). rTMS demonstrated a marked improvement in CRS-R scores (MD 3.00) and GCS scores (MD 2.92), while multisensory stimulation showed substantial benefits in GCS (MD 2.28) and GOS (MD 1.11). Acupuncture also showed strong efficacy across multiple metrics, including GCS, GOS, and Efficacy Rate. These pooled results strengthen the position of these modalities as first-line considerations in clinical protocols by providing higher-level, quantitative summaries of their benefits. Additionally, TNS, family-centered sensory-affective stimulation, and RMNS show promising therapeutic potential, though further validation is required to establish their clinical applicability and evidence strength.

In clinical efficacy assessments, while the TNS group exhibited a significantly greater improvement in GCS scores compared to the sham group (*P* = 0.028), its effect on CRS-R scores—though numerically superior—did not achieve statistical significance. This discrepancy may stem from several limitations, including the relatively small sample size of patients with post-TBI disorders of consciousness and the study's methodological constraints. Consequently, further large-scale, high-quality trials are warranted to confirm the therapeutic potential of TNS.

Evidence indicates that multisensory stimulation nursing significantly enhances clinical recovery in patients with DoC. Notably, family-involved stimulation introduces an affective dimension beyond conventional sensory input that could further facilitate consciousness recovery (28, 40–42). However, a comparative analysis reveals an intriguing discrepancy: While family-centered affective stimulation outperforms both standard care and nurse-administered interventions, its effect size on GCS improvement appears smaller than that of structured multisensory stimulation. This inconsistency may stem from heterogeneity across included studies, suggesting that the additive benefits of emotional stimulation require further validation through rigorously controlled trials.

Current evidence suggests that both right-sided and bilateral MNS demonstrate efficacy in promoting consciousness recovery. Theoretical considerations have posited that RMNS might yield superior clinical outcomes due to its more extensive cortical projections within sensorimotor areas (25, 38, 43). However, our meta-analytic findings present a paradoxical result: while both MNS and RMNS showed statistically significant efficacy, the effect size for RMNS was unexpectedly smaller than that of conventional MNS. This apparent contradiction with prior research may be attributed to several methodological and clinical factors: 1) potential inclusion of lower-quality studies in the RMNS subgroup analysis; 2) inadequate sample size for detecting true effect size differences; 3) significant heterogeneity in baseline patient characteristics, including etiology, disease duration, and severity levels.

Our systematic evaluation reveals that several neuromodulation approaches—including tDCS, rTMS, TNS, multi-sensory stimulation, and acupuncture therapy—demonstrate clinically meaningful benefits for patients with post-traumatic brain injury (TBI) disorders of consciousness (DoC). These findings underscore the need for future research to implement more rigorous experimental designs, including standardized patient stratification, larger multicenter cohorts, and improved methodological quality. Such advancements will enable more precise evaluation of efficacy differences between various stimulation modalities and facilitate optimal treatment protocol development for DoC patients.

## 5 Limitations

While this comprehensive analysis provides important insights into interventions for DoC induced by TBI, critical consideration is required to avoid misinterpretation of the findings. Several interconnected limitations collectively compromise the robustness, generalizability, and clinical applicability of the results of this study, as detailed below:

First, methodological heterogeneity and inconsistent study quality among included literature, coupled with a relatively small number of systematic reviews, meta-analyses, and RCTs and the exclusion of some high-quality studies due to non-target disease samples, have undermined the credibility of pooled results and the completeness of the evidence chain.

Second, the lack of direct comparative effectiveness research between different interventions precludes definitive conclusions regarding the selection of optimal therapeutic strategies for clinical practice. This is because without head-to-head comparisons, it is difficult to objectively evaluate which intervention yields superior efficacy, thus limiting the ability to provide clear clinical guidance for practitioners.

Third, the absence of standardized intervention protocols across included studies, coupled with insufficient systematic safety evaluation of all involved interventions, further undermines the reliability of the pooled conclusions and their practical reference value in clinical settings. The inconsistency in intervention protocols (e.g., varying parameters, implementation procedures) hinders the reproducibility of research results, while the lack of comprehensive safety assessments fails to fully address potential risks associated with the application of these interventions.

To address these limitations, we recommend targeted research efforts including high-quality multicenter RCTs with standardized protocols, mechanistic studies on neural recovery pathways, development of personalized therapy tools, and systematic safety assessments in future studies.

## 6 Conclusion

This comprehensive evidence synthesis demonstrates the significant therapeutic potential of neuromodulation therapies and multimodal rehabilitation interventions (sensory stimulation interventions and TCM-derived therapy) for TBI-induced DoC. Our analysis reveals that tDCS, rTMS, MNS, and multisensory stimulation constitute effective non-invasive therapeutic strategies, as evidenced by consistent improvements across standardized clinical outcome measures. The therapeutic armamentarium is further augmented by acupuncture and right median nerve RMNS. The integration of these evidence-based approaches into clinical practice promises to enhance recovery trajectories for patients with DoC, with consequent improvements in long-term functional outcomes and quality of life. Future research priorities should include the development of personalized treatment algorithms and the standardization of outcome assessment methodologies to advance the field of neurorehabilitation.

## Data Availability

The raw data supporting the conclusions of this article will be made available by the authors, without undue reservation.

## References

[1] Xia Y Li M Chen Z Fan M Pan Q Tian Y . Regulated cell death in traumatic brain injury: investigating mechanisms contributing to cognitive impairment. Cells. (2025) 14:1878. doi: 10.3390/cells14231878 41369366 PMC12690988

[2] Wiles MD. Management of traumatic brain injury: a narrative review of current evidence. Anaesthesia. (2022) 77:102–12. doi: 10.1111/anae.15608 35001375

[3] Solomon J Onigbinde S Adeniyi M Daramola O Gutierrez-Reyes C Fowowe M . Predictive N-Glycan signatures of severe traumatic brain injury in biofluids using LC–MS/MS. ACS Omega. (2025) 11:1883–97. doi: 10.1021/acsomega.5c10061 41552607 PMC12809573

[4] Staib-Lasarzik I Gölz C Bobkiewiecz W Somnuke P Sebastiani A Thal SC . Sortilin is dispensable for secondary injury processes following traumatic brain injury in mice. Heliyon. (2024) 10:e35198. doi: 10.1016/j.heliyon.2024.e35198 39170542 PMC11336488

[5] Guan B Anderson DB Chen L Feng S Zhou H. Global, regional and national burden of traumatic brain injury and spinal cord injury, 1990–2019: a systematic analysis for the global burden of disease study 2019. BMJ Open. (2023) 13:e075049. doi: 10.1136/bmjopen-2023-075049 37802626 PMC10565269

[6] Zarmer L Khan MS Islat G Alameddin H Massey M Kazui S . Traumatic brain injury: advances in diagnostic techniques and treatment modalities. J Clin Med. (2025) 14:7145. doi: 10.3390/jcm14207145 41156015 PMC12564102

[7] Rajan RK. A comprehensive review on adaptive plasticity and recovery mechanisms post-acquired brain injury. Neuroprotection. (2025) 3:226–52. doi: 10.1002/nep3.70006 41394307 PMC12699554

[8] Liu S Li X Jiang S Liu D Wang J. A review of advances in multimodal treatment strategies for chronic disorders of consciousness following severe traumatic brain injury. Int J Gen Med. (2025) 18:771–86. doi: 10.2147/IJGM.S502086 39967766 PMC11834669

[9] Squitti R Reale G Tondolo V Crescenti D Bellini S Moci M . Imbalance of essential metals in traumatic brain injury and its possible link with disorders of consciousness. Int J Mol Sci. (2023) 24:6867. doi: 10.3390/ijms24076867 37047843 PMC10095508

[10] Dutta RR Abdolmanafi S Rabizadeh A Baghbaninogourani R Mansooridara S Lopez A . Neuromodulation and disorders of consciousness: systematic review and pathophysiology. Neuromodulation. (2025) 28:380–400. doi: 10.1016/j.neurom.2024.09.003 39425733

[11] Qin X Chen X Zhao X Yao L Niu H Li K . Electroencephalogram prediction of propofol effects on neuromodulation in disorders of consciousness. Front Neurol. (2025) 16:1637647. doi: 10.3389/fneur.2025.1637647 41090031 PMC12517178

[12] Shu Z Wu J Lu J Zhang H Wang Y Li X . Effective DBS treatment improves neural information transmission of patients with disorders of consciousness: an fNIRS study. Physiol Meas. (2023) 44:125002. doi: 10.1088/1361-6579/ad14ab 38086065

[13] Bergeron D Mithani K RaguŽ M Chudy D Huang Y Hadjinicolaou A . Central thalamic deep brain stimulation for disorders of consciousness: an individual participant data meta-analysis. J Neurosurg. (2025) 143:1217–26. doi: 10.3171/2025.3.JNS241092 40680302

[14] Holewijn RA Wiggerts Y Bot M Verbaan D de Bie RMA Schuurman R . Surgical complications in subthalamic nucleus deep brain stimulation for parkinson's disease: experience in 800 patients. Stereotact Funct Neurosurg. (2024) 102:275–83. doi: 10.1159/000539483 38934196 PMC11457978

[15] Weaver J A Watters K Cogan A M. Interventions facilitating recovery of consciousness following traumatic brain injury: a systematic review. OTJR. (2023) 43:322–36. doi: 10.1177/15394492221117779 36047664

[16] Yao S Wang X Sun J Guo P. Efficacy of non-invasive brain stimulation for post-stroke dysphagia: a meta-analysis. Psychogeriatrics. (2024) 24:433–42. doi: 10.1111/psyg.13090 38337190

[17] Premaratne S Zoghi M Antonic-Baker A Chen Z Chen L Hamer R . Non-invasive neuromodulation for the treatment of drug-resistant epilepsy: Protocol for a systematic review and meta-analysis investigating efficacy, safety, and optimal stimulation parameters. Syst Rev. (2025) 14:214. doi: 10.1186/s13643-025-02981-2 41199344 PMC12593945

[18] Tan L Zeng L Wang N Deng M Chen Y Ma T . Acupuncture to promote recovery of disorder of consciousness after traumatic brain injury: a systematic review and meta-analysis. Evid Based Complement Alternat Med. (2019) 2019:1–14. doi: 10.1155/2019/5190515 31015851 PMC6444240

[19] Yihang G Runna M Hongjuan W Xiaoli Z Jing L Hui C . Meta-analysis of the awakening effect of multisensory stimulation in coma patients with stroke. Nurs Pract Res. (2023) 20:3027–35.

[20] Pan C Xiuqin S Shuang Q Lihua Z Min W Fang L . Meta-analysis of the efficacy of multisensory stimulation on promoting recovery in coma patients with brain injury. Chin Evid Based Nurs. (2022) 8:1725–9.

[21] Castro P Hoffner G Uhrig L Sitt JD Dehaene S Naccache L . Transcranial direct current stimulation modulates primate brain dynamics across states of consciousness. eLife. (2025) 13:e101688. doi: 10.7554/eLife.101688.3 41081761 PMC12517689

[22] Li J Fang H Liu T Wang Y Zhang X Chen L . Acupuncture for arousal combined with repetitive transcranial magnetic stimulation promotes functional reorganization of brain regions in patients with a minimally conscious state: study protocol for a randomized controlled trial. Front Neurol. (2025) 16:1610462. doi: 10.3389/fneur.2025.1610462 40832150 PMC12358844

[23] Sharma N Chahal A Rai RH Wójcik BM Alfaifi BJ Vajrala KR . Effects of non-invasive brain stimulation on arousal and alertness among traumatic brain injury patients with disorders of consciousness or persistent vegetative State: a systematic review. Acta Neurol Belg. (2025) 125:661–78. doi: 10.1007/s13760-025-02794-z 40301279

[24] Xiong Q Le K Tang Y Zhang L Wang J Chen Z . Effect of single and combined median nerve stimulation and repetitive transcranial magnetic stimulation in patients with prolonged disorders of consciousness: a prospective, randomized, single-blinded, controlled trial. Front Aging Neurosci. (2023) 15:1112768. doi: 10.3389/fnagi.2023.1112768 37168716 PMC10164991

[25] Wang P Cao W Zhou H Li X Zhang Y Liu J . Efficacy of median nerve electrical stimulation on the recovery of patients with consciousness disorders: a systematic review and meta-analysis. J Int Med Res. (2022) 50:03000605221134467. doi: 10.1177/03000605221134467 36448965 PMC9720824

[26] Liu Z Zhang X Yu B Wang Y Chen J Li H . Effectiveness on level of consciousness of non-invasive neuromodulation therapy in patients with disorders of consciousness: a systematic review and meta-analysis. Front Hum Neurosci. (2023) 17:1129254. doi: 10.3389/fnhum.2023.1129254 37292582 PMC10246452

[27] Zhang Q Ma H Huo L Wang S Yang Q Ye Z . Neural mechanism of trigeminal nerve stimulation recovering defensive arousal responses in traumatic brain injury. Theranostics. (2025) 15:2315–37. doi: 10.7150/thno.106323 39990219 PMC11840743

[28] Zuo J Tao Y Liu M Zhang X Li Y Chen H . The effect of family-centered sensory and affective stimulation on comatose patients with traumatic brain injury: a systematic review and meta-analysis. Int J Nurs Stud. (2021) 115:10384. doi: 10.1016/j.ijnurstu.2020.103846 33485101

[29] Moher D Liberati A Tetzlaff J Altman DG . Preferred reporting items for systematic reviews and meta-analyses: the PRISMA statement. PLoS Med. (2009) 6:e1000097. doi: 10.1371/journal.pmed.1000097 19621072 PMC2707599

[30] Yang X Li Y Liu Q. INPLASY202480015. 2024.

[31] Shea BJ Reeves BC Wells G Thuku M Hamel C Moran J . AMSTAR 2: a critical appraisal tool for systematic reviews that include randomised or non-randomised studies of healthcare interventions, or both. Bmj. (2017) 358:j4008. doi: 10.1136/bmj.j4008 28935701 PMC5833365

[32] Li Y Li L Huang H. Effect of non-invasive brain stimulation on conscious disorder in patients after brain injury: a network meta-analysis. Neurol Sci. (2023) 44:2311–27. doi: 10.1007/s10072-023-06743-7 36943589

[33] Wijdicks E F. Clinical scales for comatose patients: the Glasgow Coma Scale in historical context and the new FOUR Score. Rev Neurol Dis. (2006) 3:109–17. 17047576

[34] Ma H Fan S Xu Z Wan X Yang Q Yin Y . Trigeminal nerve stimulation for prolonged disorders of consciousness: A randomized double-blind sham-controlled study. Brain Stimul. (2023) 16:819–27. doi: 10.1016/j.brs.2023.05.002 37182683

[35] Giacino J T Kalmar K Whyte J. The JFK Coma Recovery Scale-Revised: Measurement characteristics and diagnostic utility11No commercial party having a direct financial interest in the results of the research supporting this article has or will confer a benefit upon the authors or upon any organization with which the authors are associated. Arch Phys Med Rehabil. (2004) 85:2020–9. doi: 10.1016/j.apmr.2004.02.033 15605342

[36] King J T Carlier P M Marion D W. Early glasgow outcome scale scores predict long-term functional outcome in patients with severe traumatic brain injury. J Neurotrauma. (2005) 22:947–54. doi: 10.1089/neu.2005.22.947 16156710

[37] Ren S Chen Y Liu Y Lv Q Peng J Song L . Acupuncture for somatosensory deficits after stroke: a systematic review and meta-analysis. Frontiers in medicine. (2025) 12:1504215. doi: 10.3389/fmed.2025.1504215 39981076 PMC11841453

[38] Wu X Xie L Lei J Yao J Li J Ruan L . Acute traumatic coma awakening by right median nerve electrical stimulation: a randomised controlled trial. Intensive Care Med. (2023) 49:633–44. 37178149 10.1007/s00134-023-07072-1 PMC10182548

[39] Yanhong S Xiuqin S Zhen F. A meta-analysis of median electrical nerve stimulation and conventional therapy in coma patients. Chin J Rehabil Med. (2017) 32:1273–7.

[40] Zhang J Jiang L Lian J. Effect of stimulating Neiguan combined with multi-sensory stimulation mode on sleep structure of patients with disturbance of consciousness after cerebral hemorrhage. World J Sleep Medne. (2025) 12:1491-4.

[41] Li W Wang L Xu C Zhang Y Chen H . Effect of multi-sensory awakening nursing model on awakening and neurological function in patients with disturbance of consciousness. Chin Med J. (2024) 59:1329-32.

[42] Norwood MF Lakhani A Watling DP Marsh CH Zeeman H. Efficacy of multimodal sensory therapy in adult acquired brain injury: a systematic review. Neuropsychol Rev. (2022) 33:693–713. doi: 10.1007/s11065-022-09560-5 36056243 PMC10769951

[43] Gu C Shang H Kong Y Peng M Jiang H Wang S . Safety of median nerve electrical stimulation in disorders of consciousness: A systematic review and meta-analysis of randomized controlled trials. PLoS ONE. (2025) 20:e0324046. doi: 10.1371/journal.pone.0324046 40743130 PMC12312889

